# The complete mitochondrial genomes of two ghost moths, *Thitarodes renzhiensis* and *Thitarodes yunnanensis*: the ancestral gene arrangement in Lepidoptera

**DOI:** 10.1186/1471-2164-13-276

**Published:** 2012-06-22

**Authors:** Yong-Qiang Cao, Chuan Ma, Ji-Yue Chen, Da-Rong Yang

**Affiliations:** 1 Key Laboratory of Tropical Forest Ecology, Xishuangbanna Tropical Botanical Garden, Chinese Academy of Sciences, Kunming, 650223, China; 2 Graduate University of the Chinese Academy of Sciences, Beijing, 100049, China; 3 State Key Laboratory of Integrated Management of Pest Insects and Rodents, Institute of Zoology, Chinese Academy of Sciences, Beijing, 100101, China

**Keywords:** *Thitarodes renzhiensis*, *Thitarodes yunnanensis*, mitochondrial genome, gene rearrangement

## Abstract

**Background:**

Lepidoptera encompasses more than 160,000 described species that have been classified into 45–48 superfamilies. The previously determined Lepidoptera mitochondrial genomes (mitogenomes) are limited to six superfamilies of the lineage Ditrysia. Compared with the ancestral insect gene order, these mitogenomes all contain a tRNA rearrangement. To gain new insights into Lepidoptera mitogenome evolution, we sequenced the mitogenomes of two ghost moths that belong to the non-ditrysian lineage Hepialoidea and conducted a comparative mitogenomic analysis across Lepidoptera.

**Results:**

The mitogenomes of *Thitarodes renzhiensis* and *T*. *yunnanensis* are 16,173 bp and 15,816 bp long with an A + T content of 81.28 % and 82.34 %, respectively. Both mitogenomes include 13 protein-coding genes, 22 transfer RNA genes, 2 ribosomal RNA genes, and the A + T-rich region. Different tandem repeats in the A + T-rich region mainly account for the size difference between the two mitogenomes. All the protein-coding genes start with typical mitochondrial initiation codons, except for *cox1* (CGA) and *nad1* (TTG) in both mitogenomes. The anticodon of *trnS(AGN)* in *T. renzhiensis* and *T. yunnanensis* is UCU instead of the mostly used GCU in other sequenced Lepidoptera mitogenomes. The 1,584-bp sequence from *rrnS* to *nad2* was also determined for an unspecified ghost moth (*Thitarodes* sp.), which has no repetitive sequence in the A + T-rich region. All three *Thitarodes* species possess the ancestral gene order with *trnI*-*trnQ*-*trnM* located between the A + T-rich region and *nad2*, which is different from the gene order *trnM*-*trnI*-*trnQ* in all previously sequenced Lepidoptera species. The formerly identified conserved elements of Lepidoptera mitogenomes (i.e. the motif ‘ATAGA’ and poly-T stretch in the A + T-rich region and the long intergenic spacer upstream of *nad2*) are absent in the *Thitarodes* mitogenomes.

**Conclusion:**

The mitogenomes of *T. renzhiensis* and *T. yunnanensis* exhibit unusual features compared with the previously determined Lepidoptera mitogenomes. Their ancestral gene order indicates that the tRNA rearrangement event(s) likely occurred after Hepialoidea diverged from other lepidopteran lineages. Characterization of the two ghost moth mitogenomes has enriched our knowledge of Lepidoptera mitogenomes and contributed to our understanding of the mechanisms underlying mitogenome evolution, especially gene rearrangements.

## Background

Insect mitogenomes are usually small closed-circular molecules (15–20 kb) containing 13 protein-coding genes (PCGs), 2 ribosomal RNA (rRNA) genes, 22 transfer RNA (tRNA) genes, and a large non-coding element termed the A + T-rich or control region [1,2]. Because of their unique features, including coding content conservation, maternal inheritance, and rapid evolution, mitogenome sequences have been widely used as molecular markers for diverse evolutionary studies [1,3]. The order of the genes in the *Drosophila yakuba* mitogenome, the first insect mitogenome to have its sequence determined, is shared by the majority of insect species, and this is therefore considered to be the ancestral order for the entire class Insecta [1,4-6]. Various gene rearrangements have been reported in other insect mitogenomes and the most common type of rearrangements involves tRNA genes [7]. All tRNA gene rearrangements can be classified as translocation, local inversion, or remote inversion (translocation and inversion) [8,9]. These rearrangements represent a molecular mitochondrial signature at the order or lower taxonomic levels [8,10,11]. Therefore, in addition to sequence data, the mitochondrial gene order can provide important evidence to establish evolutionary relationships [1,5,12]. With the increasing availability of sequence data, the mitogenome has become a model for investigating the mode and mechanism of genome evolution [13].

Lepidoptera (butterflies and moths), one of the two largest insect orders, has more than 160,000 described species that have been classified into 45–48 superfamilies [14,15]. Based on the information available in the GenBank database up to 2011, more than 41 complete or nearly complete mitogenome sequences have been determined for the Lepidoptera species. All these species are limited to six superfamilies, Tortricoidea, Bombycoidea, Noctuoidea, Pyraloidea, Geometroidea, and Papilionoidea, which belong to the lepidopteran lineage Ditrysia. Taxonomic sampling is still poor mainly because of the absence of information about the non-ditrysian lineages. A better understanding of the Lepidoptera mitogenomes requires an expansion of taxon samplings, especially of the non-ditrysian lineages (e.g. Exoporia). All the Lepidoptera mitogenomes available in GenBank are characterized by the gene order *trnMtrnItrnQ*, revealing a translocation of *trnM* compared with the ancestral gene order *trnItrnQtrnM*. It is not known whether this rearrangement is common for the whole order Lepidoptera or whether it occurred after the split of Lepidoptera. Mitogenome sequencing of non-ditrysian lineages of Lepidoptera will help address this interesting question and shed light on the underlying mechanisms of mitogenome evolution.

The ghost moth genus *Thitarodes* Viette (previously called *Hepialus*[16-19]) belongs to the family Hepialidae (Lepidoptera: Exoporia: Hepialoidea) and Hepialoidea is the most successful among the non-ditrysian lineages in terms of extant diversity [15,16]. *Thitarodes* are the only known hosts for the ascomycete *Ophiocordyceps sinensis* (Berk.), commonly known as the Chinese caterpillar fungus, which is a prized traditional Chinese medicine that is believed to boost immunity and increase stamina [20]. Members of the *Thitarodes* occupy diverse habitats on the alpine meadows in the Himalayas and on the Tibetan Plateau [21,22]. Among them, *T*. *renzhiensis* is distributed on the Renzhi and Baima Snow mountains, at altitudes of 3880–4750 m, in northwest Yunnan Province, China [23]. *Thitarodes yunnanensis* is found only on Laojun Mountain, at elevations of 3680–3750 m, in northwest Yunnan Province, China [24]. There are distinct differences in the male genital structure between these two species. Compared with *T. yunnanensis*, there is a heavily sclerotized curved spine on the end of the valve in *T. renzhiensis*[23,24].

In the present study, we sequenced the entire mitogenomes of the ghost moths, *T. renzhiensis* and *T. yunnanensis*. To confirm our findings, the 1,584-bp sequence from *rrnS* to *nad2* was also determined for a third species in *Thitarodes*. We compared the sequences with other insect mitogenomes, particularly with those of previously determined Lepidoptera species.

## Results and discussion

### Genome structure, organization, and composition

The complete mitogenomes of *T. renzhiensis* and *T. yunnanensis* are circular molecules with 16,173 bp and 15,816 bp, respectively. The two mitogenome sequences have been deposited in GenBank (*T. renzhiensis* [GenBank: HM744694] and *T. yunnanensis* [GenBank: HM744695]). Because of expansion of the A + T-rich region, the mitogenome sequence of *T. renzhiensis* is longer than any other complete Lepidoptera mitogenome sequenced to date. The 1,584-bp mtDNA sequence comprising partial *rrnS*, the A + T-rich region, *trnI*, *trnQ*, *trnM*, and partial *nad2*, was also determined for *Thitarodes* sp. [GenBank: HQ883371].

**Figure 1:**
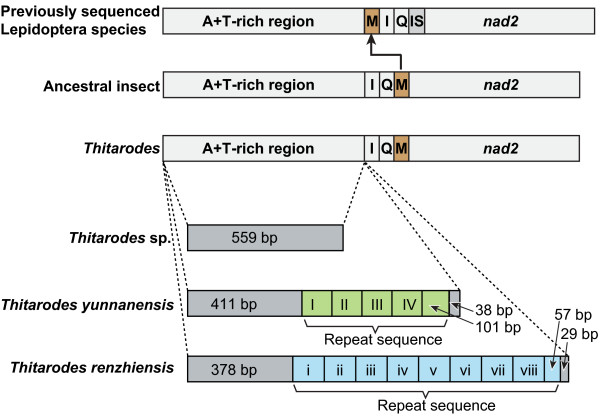
**Schematic representation of mitochondrial gene arrangements and the A + T-rich regions in three* Thitarodes *species.** All the currently determined * Thitarodes * species have the ancestral gene order. Previously sequenced lepidopteran species have the * trnM * (M) translocated upstream of *trnI* (I), *trnQ* (Q), and an intergenic spacer (IS). There are 4.9 repeat units (I–IV with 107 bp per unit) in *T. yunnanensis* and 8.5 repeat units (i–viii with 113 bp per unit) in *T. renzhiensis*. Similar repetitive sequence is absent in *Thitarodes* sp.

The *T. renzhiensis* and *T. yunnanensis* mitogenomes both include the entire set of 37 genes (13 PCGs, 22 tRNA genes, and 2 rRNA genes; see Table 1) and the A + T-rich region that is usually present in animal mitogenomes [1]. The order of the genes in the sequences of the three *Thitarodes* species was identical to that of the inferred ancestral insects, but different from the gene order in all other Lepidoptera mitogenomes sequenced to date because of the rearrangement of three tRNA genes between the A + T-region and *nad2* (Figure 1).

**Table 1:** **Annotations for the* Thitarodes renzhiensis *(*T r*) and* Thitarodes yunnanensis *(*T y*) mitogenomes**

**Gene**	**Strand**	**Gene position**		**Initiation codon/Stop codon**		**Anticodon**
		***T r***	***T y***	***T r***	***T y***	
*trnI*	J	1-65	1-67			GAT
*trnQ*	N	66-134	95-163			TTG
*trnM*	J	139-208	169-238			CAT
*nad2*	J	209-1226	239-1258	ATT/T--	ATT/TAA	
*trnW*	J	1227-1292	1260-1326			TCA
*trnC*	N	1285-1351	1319-1385			GCA
*trnY*	N	1358-1424	1391-1457			GTA
*cox1*	J	1427-2957	1460-2990	CGA/T--	CGA/T--	
*trnL(UUR)*	J	2958-3026	2991-3059			TAA
*cox2*	J	3028-3709	3062-3743	ATG/T--	ATG/T--	
*trnK*	J	3710-3780	3744-3814			CTT
*trnD*	J	3780-3844	3814-3879			GTC
*atp8*	J	3845-4006	3880-4041	ATA/TAA	ATA/TAA	
*atp6*	J	4000-4676	4035-4711	ATG/TA-	ATG/TA-	
*cox3*	J	4677-5465	4712-5500	ATG/TAA	ATG/TAA	
*trnG*	J	5468-5533	5503-5568			TCC
*nad3*	J	5534-5885	5569-5920	ATT/T--	ATA/T--	
*trnA*	J	5886-5954	5921-5989			TGC
*trnR*	J	5958-6023	5993-6058			TCG
*trnN*	J	6028-6093	6067-6132			GTT
*trnS(AGN)*	J	6094-6153	6133-6193			TCT
*trnE*	J	6154-6218	6194-6259			TTC
*trnF*	N	6221-6286	6262-6330			GAA
*nad5*	N	6287-8024	6331-8068	ATT/T--	ATT/T--	
*trnH*	N	8025-8091	8069-8135			GTG
*nad4*	N	8093-9433	8137-9477	ATG/TAA	ATG/TAA	
*nad4L*	N	9434-9726	9478-9770	ATG/TA-	ATG/TA-	
*trnT*	J	9729-9794	9773-9838			TGT
*trnP*	N	9795-9858	9839-9903			TGG
*nad6*	J	9861-10384	9906-10429	ATA/TA-	ATA/TA-	
*cob*	J	10385-11530	10430-11575	ATG/TAA	ATG/TAA	
*trnS(UCN)*	J	11536-11608	11580-11650			TGA
*nad1*	N	11624-12556	11666-12598	TTG/TAA	TTG/TAA	
*trnL(CUN)*	N	12557-12627	12599-12667			TAG
*rrnL*	N	12628-13962	12668-13996			
*trnV*	N	13963-14027	13997-14061			TAC
*rrnS*	N	14028-14806	14062-14838			
A + T-rich region	J	14807-16173	14839-15816			

J and N refer to the majority and minority strand, respectively. Position numbers refer to positions on the majority strand.

In addition to the A + T-rich region, a total of 49-bp and 81-bp noncoding sequences are present in the mitogenomes of *T. renzhiensis* and *T. yunnanensis*, respectively. In the *T. yunnanensis* mitogenome, *trnI* and *trnQ* are separated by a 27-bp intergenic spacer (ATTTT)_3_CTTTTTCAACTA whereas there is no such intergenic spacer in *T. renzhiensis*. There is a 15-bp intergenic spacer between *trnS(UCN)* and *nad1* in both mitogenomes. In this region, a conserved motif ATACTAA is present in all previously sequenced Lepidoptera mitogenomes [25-27], while the corresponding sequence is ATACTAT in *T. renzhiensis* and ATACTAC in *T. yunnanensis*.

Like other insect mitogenomes, the two newly sequenced mitogenomes contain overlapping genes. A total of 16-bp overlapping sequences occupy the same three locations in each of the two mitogenomes. One 8-bp overlap is located between *trnW* and *trnC* oriented on opposite strands; the other two locations are between *atp8* and *atp6* (7 bp) and between *trnK* and *trnD* (1 bp) on the majority strand (Table.1).

**Figure 2:**
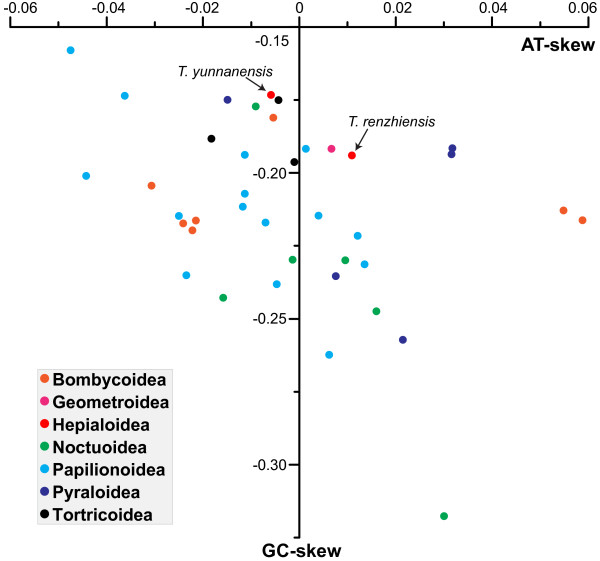
**Scatter plot of AT- and GC-skews in the lepidopteran superfamilies.** Values were calculated for the majority strand of the full-length mitogenome sequences. All the species that are represented are listed in Table 3. AT-skew = (A-T)/(A + T); GC-skew = (G-C)/(G + C).

The nucleotide compositions of the two *Thitarodes* mitogenomes are significantly biased toward A and T. The A + T content of the majority strand in *T. yunnanensis* is 82.34 %, higher than that of *T. renzhiensis* (81.28 %; see Table 2). These values fall within the range of the A + T content for other Lepidoptera species; from 77.84 % in *Ochrogaster lunifer* to 82.66 % in *Coreana raphaelis*[26,28,29]. The nucleotide skew statistics for the entire majority strand of *T. renzhiensis* (AT-skew = 0.011, GC-skew = −0.194) and *T. yunnanensis* (AT-skew = −0.006, GC-skew = −0.173) indicate slight A or T skews and a moderate C skew. A similar trend has been observed in other Lepidoptera mitogenomes (Figure 2); the AT-skew ranges from −0.04742 (*C. raphaelis*) to 0.05878 (*Bombyx mori*) and the GC-skew is always negative varying from −0.31769 (*O. lunifer*) to −0.15802 (*C. raphaelis*).

**Table 2:** **Nucleotide compositions of the* Thitarodes renzhiensis *(*T r*) and* Thitarodes yunnanensis *(*T y*) mitogenomes**

**Feature**	**A (%)**		**C (%)**		**G (%)**		**T (%)**		**A + T (%)**	
	***T r***	***T y***	***T r***	***T y***	***T r***	***T y***	***T r***	***T y***	***T r***	***T y***
Whole genome	41.09	40.93	11.17	10.36	7.54	7.30	40.20	41.41	81.28	82.34
Protein-coding genes	34.15	35.04	10.14	9.24	10.87	10.18	44.84	45.54	78.99	80.58
1st codon positions	37.18	36.94	9.54	8.90	15.62	15.75	37.66	38.41	74.84	75.35
2nd codon positions	22.07	22.10	15.99	15.91	13.12	12.90	48.82	49.09	70.89	71.18
3rd codon positions	43.20	46.10	4.89	2.90	3.87	1.88	48.04	49.11	91.24	95.22
tRNA genes	43.25	43.06	7.47	6.97	9.10	7.81	40.19	42.16	83.44	85.22
*rrnL* gene	44.34	44.92	5.32	5.04	9.29	8.95	41.05	41.08	85.39	86.00
*rrnS* gene	43.90	45.05	4.88	4.89	9.76	9.01	41.46	41.06	85.37	86.10
A + T-rich region	45.06	37.42	4.75	5.42	4.68	5.21	45.50	51.94	90.56	89.37

For PCGs, all stop codons were excluded from analysis.

### Transfer and ribosomal RNA genes

**Figure 3:**
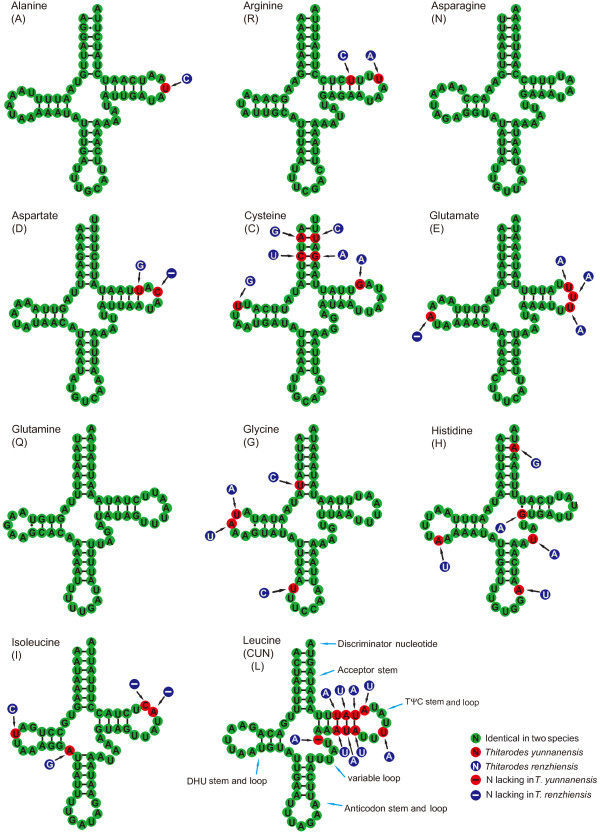
**Secondary structures of the* trnA *–* trnL(CUN) *in* Thitarodes yunnanensis *and* Thitarodes renzhiensis *mitogenomes.**

**Figure 4:**
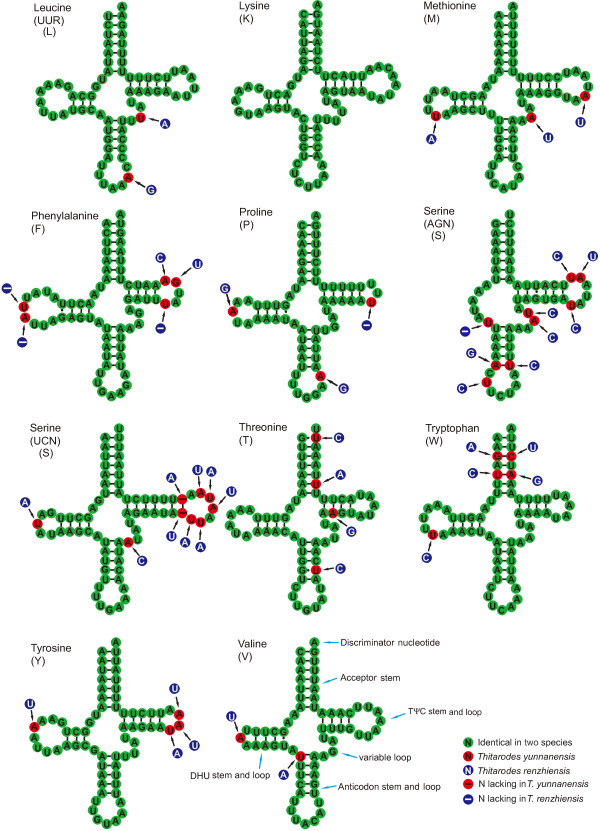
**Secondary structures of the* trnL(UUR) *–* trnV *in* Thitarodes yunnanensis *and* Thitarodes renzhiensis *mitogenomes**

The two mitogenomes have the complete set of 22 tRNA genes (Table 1) that are present in most metazoan mitogenomes. The predicted cloverleaf structures for the tRNA genes are presented in Figures 3 and 4. All tRNA genes were determined by tRNAscan-SE 1.21 [30] and the program ARWEN [31] except for *trnS(AGN)* in *T. yunnanensis*. The *trnS(AGN)* could not form the typical cloverleaf structure, because the dihydrouridine (DHU) arm is replaced by an unpaired stretch of 5 and 6 nucleotides in *T. renzhiensis* and *T. yunnanensis*, respectively. This feature is common to many arthropod mitogenomes [12,32]. The *trnS(AGN)* in *T. yunnanensis*, therefore, was determined by comparison with that of *T. renzhiensis* and previously determined Lepidoptera mitogenomes. The *trnQtrnK*, and *trnN* genes each have an identical sequence between *T. yunnanensis* and *T. renzhiensis*. A total of 39 mismatched base pairs and G-U wobble pairs are located in the acceptor (10), DHU (12), TψC (7), and anticodon (10) stems of the tRNA secondary structures in the two mitogenomes. In *T. renzhiensis*, 11 mismatched base pairs (2 A-A, 3 A-C, 1 C-U, and 5 U-U) and 10 G-U wobble pairs have been identified in 16 tRNA genes; in *T. yunnanensis*, 1 A-C, 1 C-U, and 5 U-U mismatched pairs as well as 11 G-U wobble pairs occur in 15 tRNA genes. As in the Korean hairstreak *C. raphaelis*[28], the anticodon of *trnS(AGN)* in *T. renzhiensis* and *T. yunnanensis* is UCU instead of GCU that is present in other sequenced Lepidoptera mitogenomes. The anticodons of the remaining tRNAs are each identical to those of all other available Lepidoptera mitogenomes.

Various gene orders have been found in holometabolous insect mitogenomes, and tRNA genes have frequently been involved in mitogenome rearrangements [6,25,33-36]. In the ancestral type, the tRNA gene order between the A + T-rich region and *nad2* is *trnItrnQtrnM*[1,3,6]. In all previously sequenced Lepidoptera mitogenomes, the order is *trnMtrnItrnQ*, implying that translocation of *trnM* has taken place [11,25,26]. By contrast, the present study revealed the ancestral gene arrangement in *T. renzhiensisT. yunnanensis*, and *Thitarodes* sp.. Therefore, the translocation of *trnM* is not a mitochondrial signature for the whole Lepidoptera order. Rather, the translocation event(s) likely occurred after Hepialoidea diverged from other lepidopteran lineages.

The tRNA rearrangements are generally considered to be a consequence of tandem duplication of part of mitogenome, followed by random and/or nonrandom loss of duplicated copies [37-39]. Similar non-coding sequences, ranging from 40 bp in *Parnassius bremeri*[40] to 87 bp in *Sasakia charonda* (GenBank: NC014223), are present at the position originally occupied by the transposed *trnM* in all previously sequenced Lepidoptera species but not in the three currently reported *Thitarodes* species. Because these intergenic sequences have similar lengths to those of typical tRNA genes, they were presumed to be remnants of the *trnM* gene and its boundary sequences [41]. If this is the case, the tandem duplication of mtDNA sequences including *trnM* followed by loss could be responsible for the tRNA rearrangement patterns in previously sequenced Lepidoptera mitogenomes. However, the intergenic sequences have also been considered to be products of a partial duplication of the neighboring *nad2* because of the high levels of sequence identities (up to 74 % in *Eumenis autonoe*) between these intergenic sequences and *nad2*[40,42]. If so, the duplication of partial *nad2* should be a relatively recent event considering the high sequence identities. However, low identities (<40 %) between the intergenic sequences and *nad2* were also detected in other sequenced Lepidoptera mitogenomes [40]. We should note that the short length (40–87 bp) and high A + T content (76.60–98.18 %) of the intergenic sequences make it difficult to determine the origin of the intergenic sequences based solely on sequence identities. Therefore, cautions should be taken before definite conclusions regarding the source of the intergenic sequences are drawn. Regardless of the origin of the intergenic sequences, the most likely mechanism for the tRNA rearrangement in the previously sequenced Lepidoptera species is through a tandem duplication of the region that includes *trnItrnQ*, and *trnM*, followed by losses of the supernumerary genes. Clearly, more work is required in future studies to test this assumption.

The ribosomal RNA genes (*rrnL* and *rrnS*) are located between *trnL(CUN)* and *trnV*, and between *trnV* and the A + T-rich region, respectively. The *rrnL* gene is 1,335 bp long in *T. renzhiensis* and 1,329 bp in *T. yunnanensis*; their A + T content is 85.39 % and 86.00 %, respectively (Table 2). In *T. renzhiensis*, *rrnS* is 779 bp long and the A + T content is 85.37 %; in *T. yunnanensis*, it is 777 bp long with an A + T content of 86.10 % (Table 2).

### Protein-coding genes

The mitogenomes of *T. renzhiensis* and *T. yunnanensis* contain the 13 PCGs that are usually present in animal mitogenomes and they are arranged along the mitogenome in congruence with the standard order in insects [1]. In *T. renzhiensis*, the start codons for *cox1* and *nad1* are CGA and TTG, respectively; the other PCGs start with the typical ATN codons, three (*nad2nad3*, and *nad5*) with ATT, two (*atp8* and *nad6*) with ATA, and the remainder with ATG (Table 1). Compared with *T. renzhiensisT. yunnanensis* has a different initiation codon ATA in *nad3*. Ambiguities always arise when attempting to annotate the initiation codon for *cox1* in a wide variety of species including Lepidoptera and many irregular initiation codons, including ATTACG [43], TTAG [28,44-47] and CGA [25,26,29,40,42,48-50], have been postulated for *cox1* in the sequenced Lepidoptera species. A study based on the transcript information of *Anopheles funestus* revealed that the translation initiation codon for the *cox1* gene is TCG, rather than the atypical, longer codons that had been proposed earlier [3]. Recently, expressed sequence tag data from the legume pod borer *Maruca vitrata* have shown that *cox1* may start with the CGA codon for arginine [51]. Here, we tentatively designate CGA as the *cox1* start codon, partly because this start codon has been found previously to be well conserved in 39 Lepidoptera species [40]. Further investigations are required to clarify the mechanism of *cox1* initiation in Lepidoptera. In both *T. renzhiensis* and *T. yunnanensis*, the annotated start codon of *nad1* is TTG, consistent with those in *A. funestus*[3] and *M. vitrata*[51], which were annotated based on transcript information.

The *atp8cobcox3nad1*, and *nad4* genes in *T. renzhiensis* have the conventional stop codon TAA; the remaining 8 have incomplete stop codons T or TA, 6 that are located in the genes that have tRNA genes at their 3' end, and 2 belonging to *atp6* and *nad6* upstream of *cox3* and *cob*, respectively (Table 1). The only difference between the stop codons in the two mitogenomes is for *nad2* which uses the complete stop codon TAA in *T. yunnanensis* and the incomplete stop codon T in *T. renzhiensis*. Partial stop codons are common in the mitogenomes of most insects including all sequenced Lepidoptera species [1,25,41]. The polycistronic transcript molecule is processed into mature RNA by precise endonucleolytic cleavages using the recognition signals of tRNA secondary structures; the truncated stop codons are presumed to be completed via post-transcriptional polyadenylation [52,53]. The *atp8* and *atp6* genes in the two mitogenomes have a 7-bp overlap, a common feature of Lepidoptera and many other arthropod mitogenomes [25,54].

The A + T content of the PCGs, excluding stop codons, is 78.99 % and 80.58 % in *T. renzhiensis* and *T. yunnanensis*, respectively, which is lower than the A + T content of the mitogenomes as a whole (Table 2). In *T. renzhiensis* and *T. yunnanensis*, the third codon positions have a considerably higher A + T content (91.24 % and 95.22 %, respectively) than the first and second codon positions; the strongest bias toward T is in the second codon positions (48.82 % and 49.90 %, respectively), and the lowest content of G is in the third codon positions (3.87 % and 1.88 %, respectively; Table 2).

**Figure 5:**
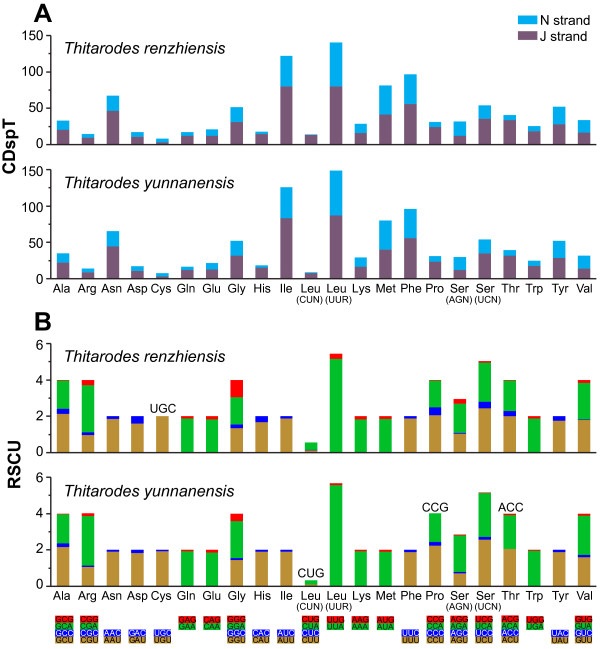
**Codon usage pattern (A) and the relative synonymous codon usage (RSCU) (B).** CDspT, codons per thousand codons. Codons that are absent in the mitogenomes are provided at the top of columns.

The codon usage bias has been detected in the currently sequenced *Thitarodes* mitogenomes. There are a total of 3720 codons, excluding stop codons, in each of the two *Thitarodes* mitogenomes. Among them, the three most abundant codons in both mitogenomes are UUA [Leu (UUR)], AUU (Ile), and UUU (Phe). As a consequence, Leu (UUR), Ile, and Phe are the three most frequently used codon families (Figure 5). The rarest used codon family is Cys. When PCGs on the majority and minority strands are considered separately, the three most abundant codon families are also Leu (UUR), Ile, and Phe (Figure 5). The usage of both four- and two-fold degenerate codons exhibits a strong A + T-bias in the third codon positions. G + C-rich codons are less preferred, with UGC absent in *T. renzhiensis*, and CUG, CCG, and ACC lost in *T. yunnanensis* (Figure 5). A similar codon usage pattern and A + T-bias in the third codon positions are present in previously sequenced Lepidoptera mitogenomes, which were analyzed and averaged for each superfamily (Additional file 1: Figure S1, Additional file 2: Figure S2).

### The A + T-rich region

The length and A + T content of the A + T-rich regions are 1,367 bp and 90.56 % in *T. renzhiensis*, 978 bp and 89.37 % in *T. yunnanensis*, and 559 bp and 92.84 % in *Thitarodes* sp.. The A + T-rich region of *T. renzhiensis* is the longest of all the sequenced Lepidoptera mitogenomes; the shortest is 319 bp in *O. lunifer*[26].

The A + T-rich region of *T*. *renzhiensis* includes a tandem repeat region consisting of eight 113-bp copies and one partial copy of a 57-bp sequence (Figure 1). In *T*. *yunnanensis*, the A + T-rich region includes four complete repeat units (107 bp) and one truncated repeat unit (101 bp). However, the A + T-rich region of *Thitarodes* sp. consists entirely of non-repetitive sequences (Figure 1). The conspicuous macrorepeat units (>50 bp long) commonly found in other insects are also present in previously sequenced Lepidoptera mitogenomes, for example, *Bombyx mandarina* (126 bp) [55], *Papilio maraho* (252 bp) [56], and *Spilonota lechriaspis* (124 bp) [57]. An explanation for the origin of these repeat sequences is slipped-strand mispairing during mtDNA replication [58,59]. These repeat sequences mainly account for length variations in Lepidoptera mitogenomes.

Downstream of the *rrnS* gene in the previously sequenced Lepidoptera mitogenomes, there is a widely conserved structure that includes the motif ‘ATAGA’ and a 16–22 bp poly-T stretch. It has been suggested that this structure might function as a signal for mtDNA replication initiation [42,60,61]. However, these conserved elements are not found in the mitogenomes of *T*. *yunnanensisT*. *renzhiensis*, and *Thitarodes* sp., indicating that these structural motifs are not conserved in the non-ditrysian Lepidoptera species. The *Thitarodes* mitogenomes may adopt a different strategy for replication initiation. The absence of the conserved elements in the A + T-rich region, together with above mentioned structures, such as the presence of the ancestral gene arrangement, and the absence of the intergenic spacer upstream of *nad2*, demonstrates that the *Thitarodes* mitogenomes have unusual features compared with the previously determined Lepidoptera mitogenomes. Therefore, in future studies, more attention should be paid to non-ditrysian lineages when comparing Lepidoptera mitogenomes.

## Conclusions

*Thitarodes renzhiensis* and *T*. *yunnanensis* mitogenomes are the first representatives of non-ditrysian lineages of Lepidoptera. The arrangement of the tRNA genes between the A + T-region and *nad2* is *trnI*-*trnQ*-*trnM*, different from those of previously sequenced Lepidoptera mitogenomes but identical to the ancestral gene order. Therefore, the previously identified tRNA rearrangement is not a synapomorphy for Lepidoptera mitogenomes. This result indicates that the tRNA rearrangement event(s) likely occurred after Hepialoidea diverged from other lepidopteran lineages. In addition, compared with other Lepidoptera mitogenomes, *T. renzhiensis* and *T. yunnanensis* mitogenomes have other unique structural characters such as the lack of the intergenic spacer upstream of *nad2*. Therefore, non-ditrysian lineages should be taken into consideration in future comparative mitogenomic studies of Lepidoptera. Characterization of the two mitogenomes has contributed to our understanding of Lepidoptera mitogenomes and provided insights into mitogenome evolution, especially gene rearrangements.

## Methods

### Specimen collection and DNA extraction

Samples of *T. renzhiensis* and *T. yunnanensis* were obtained from cultures in Diqing, Yunnan Province, China. Specimens of *Thitarodes* sp., whose complete binomial name has not been specified, were collected in the Qilian Mountain, Qinghai Province, China. All the specimens were preserved in anhydrous ethanol and stored at −20 °C until used for DNA extraction. Whole genomic DNA was extracted from an ethanol-preserved larva with the DNeasy Blood & Tissue kit (QIAGEN, Valencia, California, USA). DNA quality was assessed by electrophoresis in a 1 % agarose gel stained with ethidium bromide.

### PCR amplification and sequencing

The entire mitogenomes of both *T. renzhiensis* and *T. yunnanensis* were amplified in 10 overlapping fragments (F1 to F10) using long PCR. All primers were designed based on the conserved nucleotide sequences of the known Lepidoptera mitogenome sequences (Table 3). Primer sequences and locations for each long PCR are listed in Additional file 3: Table S1. The fragments were amplified using LA Taq (TaKaRa Co., Dalian, China) with an initial denaturation at 95 °C for 2 min, followed by 30 cycles at 95 °C for 30 s, annealing at 48–60 °C for 45 s, and extension at 68 °C for 3–5 min, with a final elongation at 70 °C for 7–10 min after the last cycle. All the amplified products were sequenced directly except for the F10 which was sequenced after being cloned into pGEM-T Easy Vector (TianGen Biotech Co., Beijing, China). For each PCR product, the full double-stranded sequence was determined by primer walking.

**Table 3:** List of taxa analyzed in this study

**Superfamily**	**Family**	**Species**	**GenBank ID**	**References**
Bombycoidea	Bombycidae	*Bombyx mandarina*	FJ384796	[47]
		*Bombyx mori*	AY048187	[62]
	Saturniidae	*Antheraea pernyi*	NC004622	[45]
		*Antheraea yamamai*	NC012739	[46]
		*Eriogyna pyretorum*	NC012727	[29]
		*Saturnia boisduvalii*	NC010613	[63]
	Sphingidae	*Manduca sexta*	NC010266	[25]
Geometroidea	Geometridae	*Phthonandria atrilineata*	NC010522	[49]
Hepialoidea	Hepialidae	***Thitarodes renzhiensis***	**HM744694**	**This study**
		***Thitarodes yunnanensis***	**HM744695**	**This study**
Noctuoidea	Arctiidae	*Hyphantria cunea*	GU592049	[48]
	Lymantriidae	*Lymantria dispar*	NC012893	[64]
	Noctuidae	*Helicoverpa armigera*	NC014668	[61]
		*Sesamia inferens*	JN039362	Unpublished
	Notodontidae	*Ochrogaster lunifer*	NC011128	[26]
		*Phalera flavescens*	JF440342	Unpublished
Papilionoidea	Lycaenidae	*Coreana raphaelis*	NC007976	[28]
		*Protantigius superans*	HQ184265	[65]
		*Spindasis takanonis*	HQ184266	[65]
	Nymphalidae	*Acraea issoria*	NC013604	[60]
		*Apatura ilia*	JF437925	Unpublished
		*Apatura metis*	NC015537	Unpublished
		*Argynnis hyperbius*	NC015988	[66]
		*Calinaga davidis*	NC015480	[67]
		*Hipparchia autonoe*	GQ868707	[42]
		*Sasakia charonda*	NC014223	Unpublished
	Papilionidae	*Papilio maraho*	FJ810212	[56]
		*Parnassius bremeri*	FJ871125	[40]
		*Teinopalpus aureus*	NC014398	Unpublished
	Pieridae	*Pieris melete*	NC010568	[50]
		*Pieris rapae*	NC015895	[68]
Pyraloidea	Crambidae	*Chilo suppressalis*	NC015612	[69]
		*Cnaphalocrocis medinalis*	NC015985	[69]
		*Diatraea saccharalis*	FJ240227	[70]
		*Ostrinia furnacalis*	NC003368	[71]
		*Ostrinia nubilalis*	NC003367	[71]
Tortricoidea	Tortricidae	*Adoxophyes honmai*	NC008141	[72]
		*Grapholita molesta*	NC014806	[73]
		*Spilonota lechriaspis*	HM204705	[57]

### Sequence assembly and annotation

Raw sequence files were proof read and assembled into contigs using ContigExpress included in the Vector NTI (9.1) package.

PCGs were determined and translated into putative proteins using the ORF finder in the DNAStar package (DNAStar Inc., Madison, WI, USA). The identity of these polypeptides was established using the BLAST program available on the NCBI web site. To determine the gene boundaries, the 5' ends of PCGs were assumed to be at the first legitimate in-frame start codon (ATN, GTG, TTG, GTT) in an open reading frame (ORF) that was not located within an upstream gene encoded on the same strand; the 3' ends were inferred to be at the first in-frame stop codon encountered downstream of the start codon. When the stop codon was located within the sequence of a downstream gene encoded on the same strand, a truncated stop codon (T or TA) adjacent to the beginning of the downstream gene was designated as the termination codon [2,11]. This codon was assumed to be completed by polyadenylation after transcript processing [26].

Identification of tRNA genes was performed using the tRNAscan-SE Search Server [30] with invertebrate mitochondrial codon predictors and the program ARWEN [31]. The predicted tRNA secondary structures were compared and manually adjusted. The *rrnL* was annotated to extend to boundaries of the flanking *trnL(CUN)* and *trnV*. The 3' end of *rrnS* was annotated to be adjacent to the start of *trnV*, while the 5' end was determined via comparison with orthologous sequences of other Lepidoptera mitogenomes using MEGA ver4.0 [74].

### Genomic analysis

Nucleotide sequences of the 13 mitochondrial PCGs of the previously determined 37 Lepidoptera species (Table 3) were downloaded from the METAMiGA database (http://amiga.cbmeg.unicamp.br/) [75]. Nucleotide compositions and codon usage (excluding stop codons) in *T. renzhiensis* and *T. yunnanensis* mitogenomes were calculated with MEGA. To measure synonymous codon usage bias, RSCU values were also computed. Average codon usage and RSCU values were further analyzed for each superfamily used in this study. GC-skew = (G-C)/(G + C) and AT-skew = (A-T)/(A + T) were used to measure base compositional differences [76]. The tandem repeats in the A + T-rich region were predicted using the Tandem Repeats Finder [77].

## Abbreviations

atp6 and atp8, ATP synthase subunits 6 and 8; cob, cytochrome b; cox1–3, cytochrome c oxidase subunits 1–3; nad1–6 and nad4L, NADH dehydrogenase subunits 1–6 and 4 L; rrnS and rrnL, small and large ribosomal RNA (rRNA) subunits; trnX, transfer RNA (tRNA) genes with X representing the one-letter abbreviation of the corresponding amino acid.

## Competing interests

The authors declare that they have no competing interests.

## Authors’ contributions

DRY conceived and designed this study. JYC collected specimens and extracted DNA. YQC carried out molecular experiments. YQC and CM analyzed the data and drafted the manuscript. DRY thoroughly revised the manuscript. All authors contributed to the manuscript and approved the final version.

## Supplementary Material

Additional file 1 **Figure S1.** Codon distribution in mitogenomes of currently used lepidopteran superfamilies. CDspT, codons per thousand codons. Click here for file

Additional file 2 **Figure S2.** The relative synonymous codon usage (RSCU) in mitogenomes of currently used lepidopteran superfamilies. Click here for file

Additional file 3 **Table S1.** PCR primer sequences. Click here for file
