# Supplementary figures and images for: The complete mitochondrial genomes of two ghost moths, Thitarodes renzhiensis and Thitarodes yunnanensis: the ancestral gene arrangement in Lepidoptera
Source: BMC Genomics. 2012 Jun 22;13:276. doi: 10.1186/1471-2164-13-276 (PMC3463433; doi:10.1186/1471-2164-13-276)

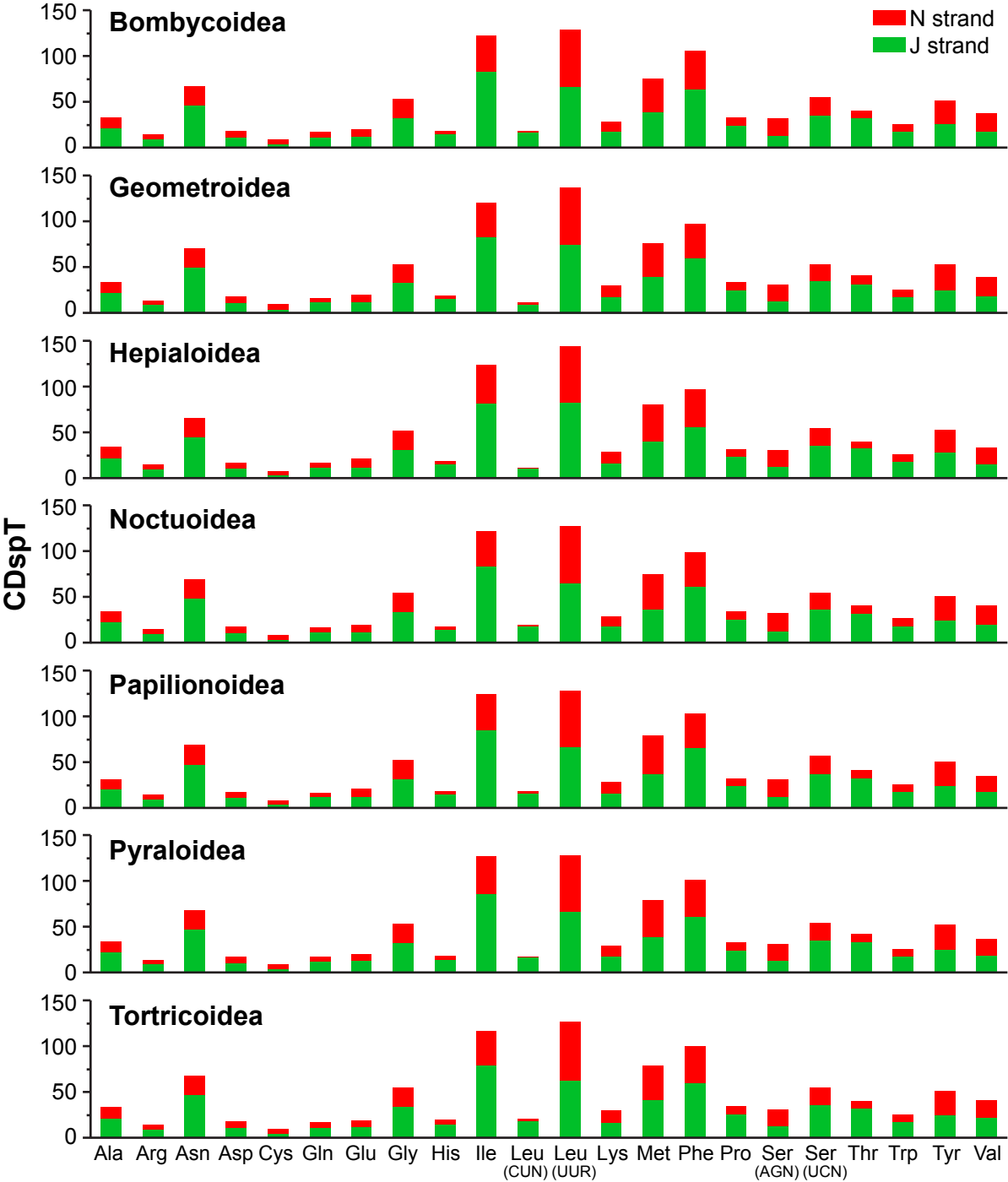

Supplement: Additional file 1 — Figure S1. Codon distribution in mitogenomes of currently used lepidopteran superfamilies. CDspT, codons per thousand codons. [file 1471-2164-13-276-S1.pdf]

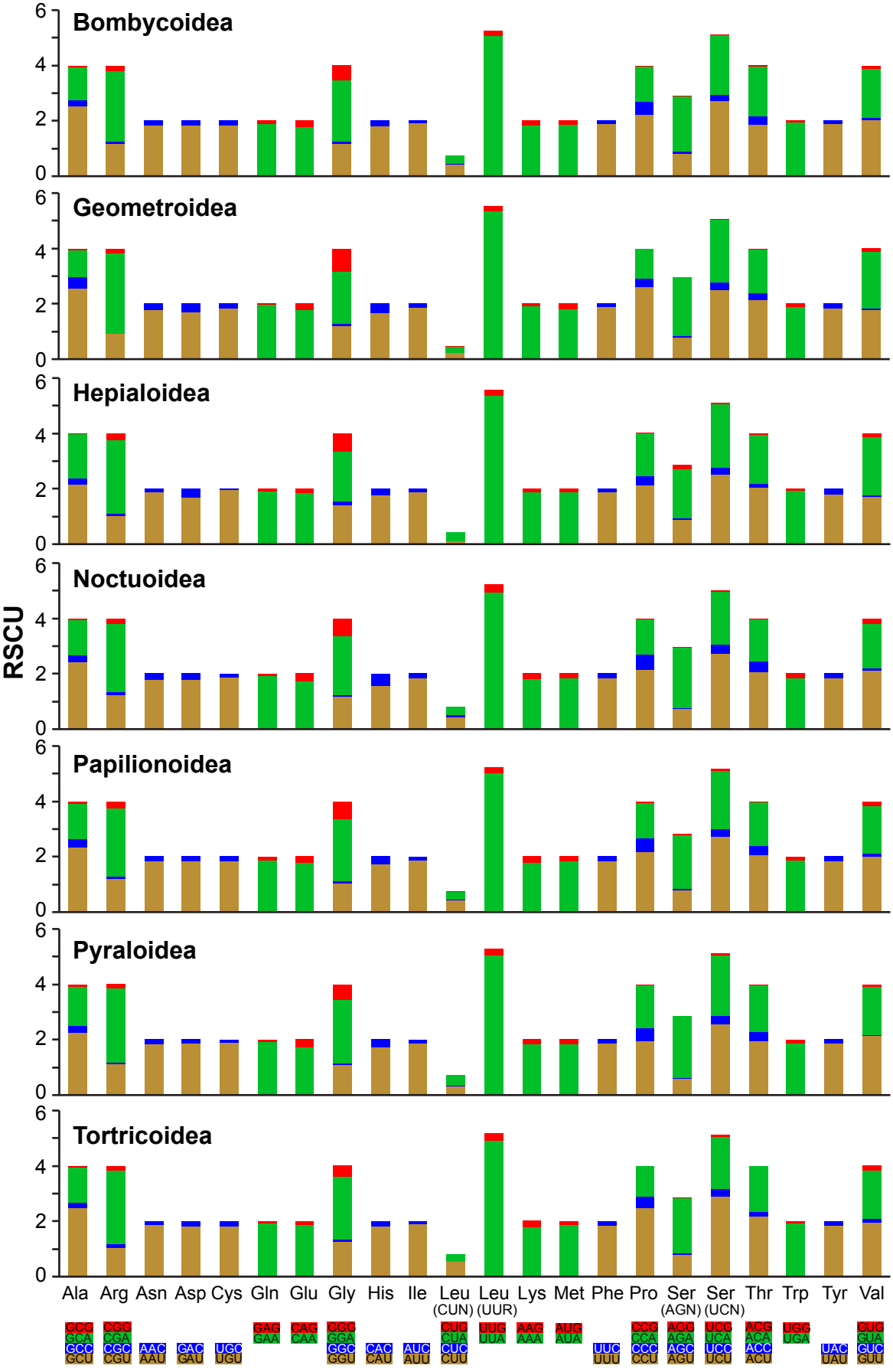

Supplement: Additional file 2 — Figure S2. The relative synonymous codon usage (RSCU) in mitogenomes of currently used lepidopteran superfamilies. [file 1471-2164-13-276-S2.pdf]
